# Gamma3 nail with U-Blade (RC) lag screw is effective with better surgical outcomes in trochanteric hip fractures

**DOI:** 10.1038/s41598-020-62980-2

**Published:** 2020-04-07

**Authors:** Seung-Beom Han, Jae-Kyun Jung, Chul-Young Jang, Dae-Kyung Kwak, Jeong-Woo Kim, Je-Hyun Yoo

**Affiliations:** 1 Department of Orthopaedic Surgery, Korea University Anam Hospital, Korea University School of Medicine, Seoul, South Korea; 2 Department of Orthopaedic Surgery, Hallym University Sacred Heart Hospital, Hallym University School of Medicine, 22 Gwanpyeong-ro 170beon-gil, Dongan-gu, Anyang 14068 South Korea

**Keywords:** Outcomes research, Risk factors

## Abstract

The objective of this retrospective study was to investigate the surgical outcomes of AO/OTA 31 A1-3 trochanteric fractures treated with the new-generation Gamma3 nail with U-Blade (RC) lag screw and to analyze the risk factors related to fixation failure. A total of 318 consecutive patients who underwent cephalomedullary nailing using Gamma3 nail with U-Blade lag screw for trochanteric hip fractures between September 2015 and June 2018 were enrolled. The average age was 80 years and most patients (69%) were women. The mean follow-up was 12.2 months with a minimum of 6 months. 309 (97.2%) showed bony union with a mean time to union of 13.5 ± 8.7 weeks. Cut-out occurred in 2 patients (0.6%) and 7 patients showed excessive collapse (≥15 mm) of the proximal fragment. These 9 patients were assigned to the failure group. The presence of a basicervical fracture component and comminution of the anterior cortex on preoperative 3-D CT showed a significant association with fixation failure, including cut-out, although comminution of the anterior cortex was the only independent risk factor for fixation failure on multivariate logistic regression analysis. Gamma3 nail with U-Blade lag screw showed favorable results for trochanteric hip fractures, with low cut-out rate (0.6%). However, more caution is required in treating trochanteric fractures with a basicervical fracture component and anterior cortex comminution even with this nail.

## Introduction

Trochanteric hip fractures are among the most common injuries in the elderly population, and their incidence continues to increase owing to demographic changes^1,2^. Successful operative treatment of these fractures is essential to enable maximum return of function in these generally debilitated elderly patients^3^. Although the best fixation method remains debated, cephalomedullary (CM) nailing has become the surgical option of choice for most surgeons, especially in unstable fracture patterns in which the implant has superior mechanical properties over extramedullary systems^3–6^. It has also been suggested that patients treated with CM nails mobilize faster and better than those treated with sliding hip screws^7^. Along with the continuous evolution of implant design to improve the rotational stability of the proximal fragment and the cut-out resistance, the Gamma nail (Stryker Trauma, Schönkirchen, Germany) and Proximal Femur Nail Antirotation (PFNA) (DePuy Synthes, Umkirch, Germany) have been the most commonly used nails for the treatment of trochanteric hip fractures, including basicervical fracture patterns. When treating these fractures even with these nails, more accurate reduction at medial and anterior cortices using tonsil clamp or bone hook and appropriate entry point of the nail and position of lag screw within the femoral head are essential to obtain favorable outcomes and avoid fixation failure as the fracture is more comminuted and unstable^8,9^.

However, fixation failures, such as cut-out, still occur owing to poor bone quality and an unstable fracture pattern despite these technical advances. Further, the incidence of comminuted unstable trochanteric fractures with a basicervical fracture component continues to increase as the osteoporotic elderly population increases^10–12^, and these fractures have been known to be a risk factor of fixation failure in CM nailing^10,13–15^. Recent studies reported a cut-out rate of between 2% and 8% in elderly patients treated with these nails^13,16,17^. To reduce fixation failures such as cut-out, the design of the nails must be taken into account in addition to patient-related characteristics, the quality of reduction, and the positioning of the lag screw. Recently, an additional U-Blade (RC) lag screw for the Gamma3 nail was introduced to provide additional rotational stability to the proximal fragment. This nail allows replacing the standard lag screw with a U-blade lag screw set (a combination of the lag screw with a U-shaped clip increasing the diameter by 2 mm). The resulting increase in surface area (by about 15%) improves the stability against rotation and cut-out, especially in unstable fracture patterns and highly osteoporotic bone^18^. However, to date, the current literature contains little information about the surgical outcomes of the use of Gamma3 nail with U-Blade lag screw in trochanteric hip fractures.

**Figure 1:**
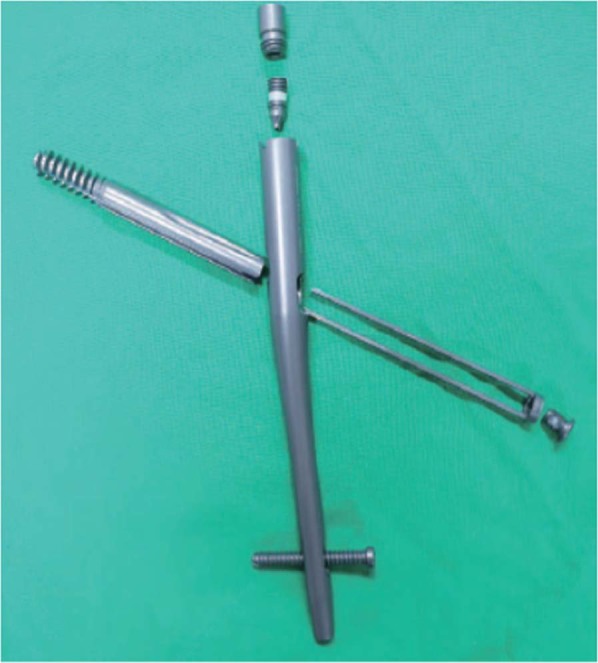
Photograph of Gamma3 nail with U-Blade (RC) lag screw.

Therefore, the objective of this retrospective study was to investigate the surgical outcomes of AO/OTA 31 A1-3 trochanteric hip fractures treated with Gamma3 nail with U-Blade lag screw (Fig. 1). In addition, we sought to evaluate the risk factors associated with fixation failures such as cut-out.

## Methods

This study was approved by the institutional review board of Hallym University Sacred Heart Hospital. The institutional review board waived the informed consent for this study owing to its retrospective nature. All methods were carried out in accordance with the relevant guidelines and regulations.

A prospectively compiled database was used to recruit patients with AO/OTA 31A1-3 trochanteric hip fractures treated with Gamma3 nail with U-Blade lag screw at two university hospitals between September 2015 and June 2018. Patients with polytrauma and pathological fractures were excluded.

**Figure 2:**
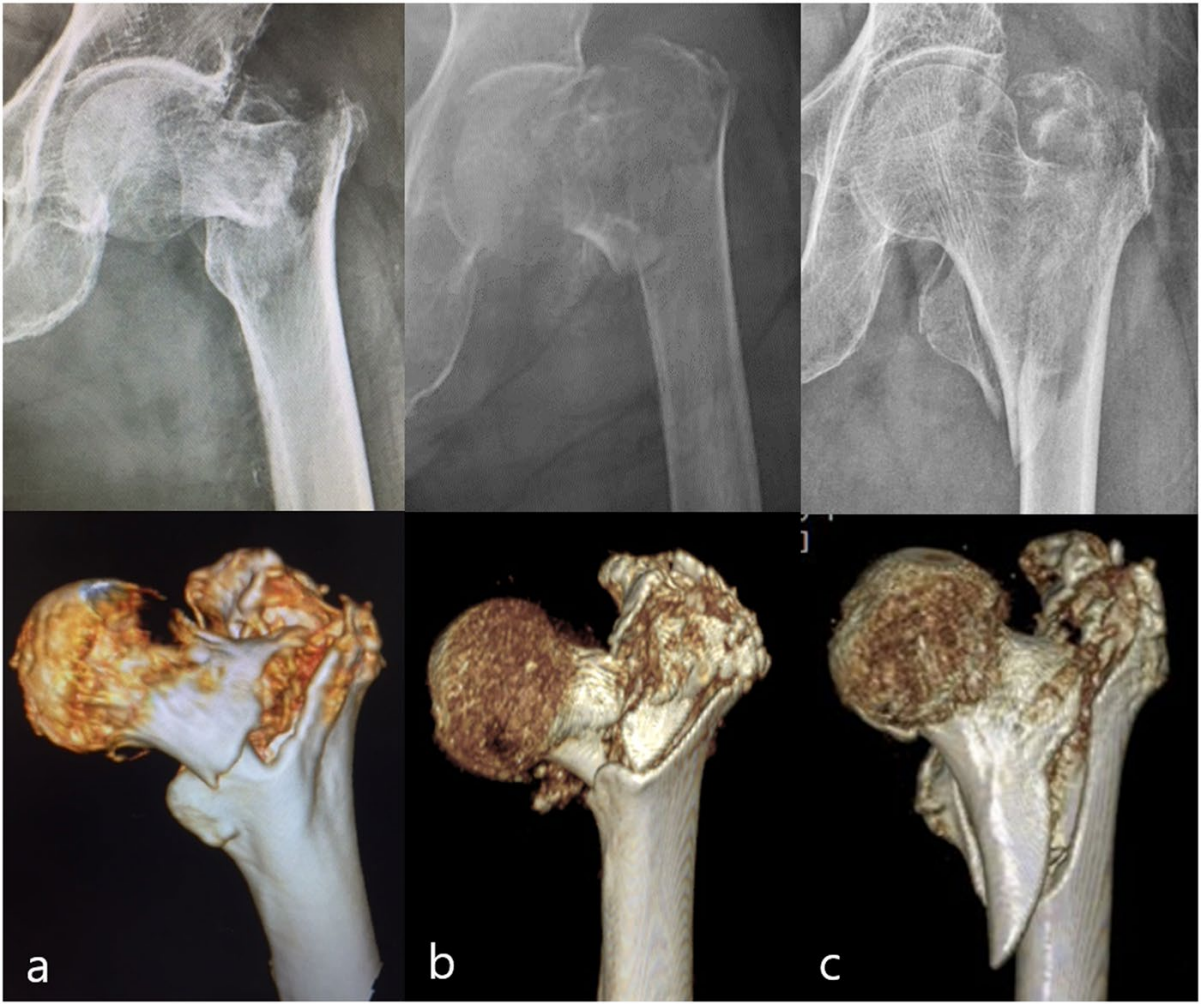
(**a)** Basicervical fracture component, (**b)** comminution of the anterior cortex, and **(c)** comminution of GT confirmed on preoperative radiographs and 3-D CTs.

Data were retrieved from the database of each department and completed through chart reviews. Standard radiographs and three-dimensional computed tomography (3-D CT) scans of each patient were taken for a detailed assessment of the fracture pattern and exact classification before surgery. All fractures were classified by two (JKJ and CYJ) of the authors using the AO/OTA system^19^. Trochanteric fractures were categorized as stable (A1) or unstable (A2, A3). In addition, the presence of a basicervical fracture component and comminution of the anterior cortex and greater trochanter (GT) were confirmed on 3-D CT by two of the authors (JKJ and CYJ) (Fig. 2). Basicervical trochanteric fracture was defined as a fracture in which the main fracture line of the proximal fragment crosses close to the base of the femoral neck and its junction with the intertrochanteric region^10–12^. The minimum follow-up was 6 months, the time at which general fracture healing was achieved. Demographic data such as gender, age, body mass index (BMI), bone mineral density (BMD), American Society of Anesthesiologists (ASA) score, and the time from admission to operation were collected from the electronic patient records. BMD was measured in the contralateral femoral neck using dual-energy x-ray absorptiometry.

All fractures were diagnosed using standard radiographs and 3-D CT. Most patients underwent index surgery under general or spinal anesthesia within 2 days and no longer than 3 days after admission to the two hospitals, with a mean time from admission to operation of 2.6 ± 2.4 days. The exceptions were specific patients with severe life-threatening systemic comorbidities, for whom a longer time was needed to optimize the medical condition through further evaluation and appropriate preoperative management. All operations were performed on the fracture table under fluoroscopic guidance, by two hip and trauma surgeons (SBH and JHY) at the two hospitals, each with an experience of >10 years in CM nailing for hip fractures. In all patients, a standard 170-mm Gamma3^®^ nail with U-Blade lag screw and one distal locking screw of appropriate length were used. A suction drain was not used in all patients. The lag screw was placed in accordance with Baumgartner *et al*.’s suggestion that the tip-apex distance (TAD) should be <25 mm to minimize the risk of cut-out and in the center-center or inferior-center position^8^.

Standing with weight bearing as tolerated and ambulation using a walker were initiated at around 2 to 3 days postoperatively depending on patients’ health condition. Patients were followed up at 3, 6, and 12 months postoperatively. Anteroposterior and lateral radiographs were obtained at each follow-up visit. Fracture union was defined as the presence of visible bone trabeculae between bone fragments in both views, accompanied by pain-free walking.

The radiographic measurements were standardized. All radiographs were calibrated with the diameter of the nail used in each case, using an electronic picture archiving and communication system (STARPACS; Infinitt Healthcare, Seoul, South Korea). On postoperative radiographs, TAD and calcar-referenced TAD (calTAD) were measured as described by Baumgartner *et al*.^8^ and Kashigar *et al*.^20^, respectively. Reduction quality was assessed using a slight modification of the criteria of Baumgartner *et al*.^21^. In addition, the position of the lag screw was assessed on anteroposterior and lateral views, and the reduction status was categorized as anatomical, extramedullary, and intramedullary. The extent of lag screw sliding was measured according to the method proposed by Paul *et al*.^22^ on anteroposterior views taken postoperatively and at the final follow-up. The TAD, calTAD, and extent of lag screw sliding were evaluated independently by two orthopedic surgery fellows (JKJ and CYJ) with significant measurement experience. Each surgeon performed two measurements for each case, with an interval of 2 weeks between measurements, and the average of the values measured was used in the analysis. The intraobserver and interobserver reliabilities were calculated and assessed using the intraclass correlation coefficient (ICC), which quantifies what proportion of the difference is due to measurement variability. The ICC can assume any value from 0 to 1, where a value greater than 0.75 represents good agreement and less than 0.40 represents poor agreement. The intraobserver reliabilities of TAD, calTAD, and lag screw sliding were 0.82, 0.81, and 0.85, respectively, and the interobserver reliabilities were 0.76, 0.77, and 0.79, respectively. Thus, the radiographic measurements for these parameters had good agreement.

All implant-related failures, such as nail breakage, penetration or cut-out of the lag screw, and excessive sliding or collapse, were reported. Cut-out was defined as projection of the lag screw ≥1 mm of the femoral head^23^. Excessive sliding of the lag screw was defined as ≥15 mm, which has been known to be associated with a high risk of fixation failure^24^.

### Statistical analyses

Basic descriptive statistical analyses were used to describe the study population. Averages or percentages of values were obtained using the statistical package SPSS version 17.0 (SPSS Inc., Chicago, IL, USA). For comparison between two groups (union group vs. failure group, non-basicervical group vs. basicervical group), Student’s t-test for continuous variables. For categorical variables, the chi-square test was used, whereas Fisher’s exact test was used when the expected counts were <5. In all analyses, statistical significance was determined by a value of p < 0.05. Multivariate logistic regression was performed to determine the predictable factors related to fixation failure and basicervical trochanteric fracture. Odds ratios (ORs) were obtained with 95% confidence intervals (CIs). ICCs were used to determine the levels of intra-rater and inter-rater agreement with respect to radiographic measurements. Landis and Koch^25^ characterized correlation coefficients of 0 to 0.20 as indicating slight agreement, 0.21 to 0.40 as fair, 0.41 to 0.60 as moderate, 0.61 to 0.80 as substantial, and 0.81 to 1 as almost perfect agreement.

## Results

During the study period, 345 patients with AO/OTA 31 A1-3 trochanteric hip fractures were identified at the two hospitals. Twenty-seven patients were lost to follow-up or had no appropriate radiological follow-up.

**Figure 3:**
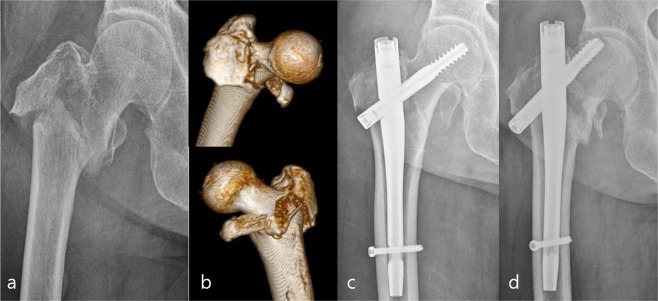
(**a)** Initial radiograph and (**b)** 3-D CTs of a 83-year-old woman showing an unstable comminuted pertrochanteric fracture with a basicervical fracture component and detached GT. (**c)** Postoperative radiograph showing good reduction status. **(d)** Anteroposterior radiograph showing cut-out of a lag screw 3 months after surgery.

Of the total 345 patients, 318 were finally enrolled in this study with a mean follow-up of 12.2 ± 6.4 months. This cohort was composed of 97 male and 221 female patients with an average age of 80.1 years (range 31–99 years) (Table 1). The radiological results for these patients were shown in Table 2. Of the 318 patients (318 fractures), 309 (97.2%) showed bony union with a mean time to union of 13.5 ± 8.7 weeks and a mean sliding distance of lag screw of 4.6 ± 4.2 mm, except for 9 patients with cut-out or excessive sliding. These 9 patients were assigned to the failure group. Cut-out occurred in 2 patients (0.6%) (Fig. 3) and excessive sliding of the proximal fragment occurred in the remaining 7 patients, with a mean time from surgery to failure of 30.8 ± 33.0 weeks.

**Table 1:** Demographic and radiographic data (n = 318).

Variables	
Age	80.1 ± 10.2
Gender ratio (male: female)	97: 221
Body mass index (kg/m^2^)	22.2 ± 3.6
Bone mineral density (T-score in femur neck)	−3.0 ± 1.1
Affected side; right: left	163: 155
**ASA score**	
II	93 (29.4)
III	196 (61.6)
IV	29 (9.0)
**Fracture type by AO/OTA classification**	
A1	150 (47.2)
A2	160 (50.3)
A3	8 (2.5)
Basicervical fracture component	158 (49.7)
Anterior cortex comminution	50 (15.7)
GT comminution	171 (53.8)

Data were presented by number (%) of patients or mean ± standard deviation.

ASA, American Society of Anesthesiologists; GT, greater trochanter.

**Table 2:** Radiological results (n = 318).

Variables	
**Lag position (anteroposterior plane)**	
Superior	6 (1.9)
Center	161 (50.6)
Inferior	151 (47.5)
**Lag position (lateral plane)**	
Anterior	68 (21.4)
Center	185 (58.2)
Posterior	65 (20.4)
TAD (mm)	13.6 ± 3.6
Cal-TAD (mm)	19.9 ± 4.2
**Quality of reduction**	
Good	242 (76.1)
Acceptable	72 (22.6)
Poor	4 (1.3)
**Reduction status**	
Anatomical	232 (73.0)
Extramedullary	55 (17.3)
Intramedullary	31 (9.7)
Sliding distance (mm)	4.6 ± 4.2

Data were presented by number (%) of patients or mean ± standard deviation.

TAD, tip-to-apex distance; Cal, calcar.

Demographic data (e.g., age, gender, BMI, BMD, and ASA grade) and perioperative data (e.g., time from admission to surgery, operation time, and type of anesthesia) showed no differences between the union group and the failure group. In terms of radiological findings, the presence of basicervical component and comminution of the anterior cortex on preoperative 3-D CT showed significant differences between the two groups, with greater proportions in the failure group (88.9% vs. 48.5%, p = 0.019 and 66.7% vs. 14.2%, p = 0.001, respectively). However, the lag screw position, TAD, calTAD, and quality and status of reduction showed no significant difference between the two groups (Table 3). On multiple logistic regression, comminution of the anterior cortex was the only independent risk factor for fixation failure (OR 9.36, p = 0.002), although the presence of a basicervical component showed a tendency to increase the risk of fixation failure (OR 5.95, p = 0.087).

**Table 3:** Comparison of demographic, clinical, and radiographic data between union and failure groups.

Variables	Union group (n = 309)	Failure group (n = 9)	p-value
Age (years)	80.0 ± 10.3	81.4 ± 6.8	0.681
Gender (female: male)	214: 95	7: 2	0.727
BMI (kg/m^2^)	22.1 ± 3.6	22.8 ± 3.2	0.596
BMD (T-score)	−3.0 ± 1.1	−2.8 ± 0.7	0.673
ASA score			0.442
II	89 (28.8)	4 (44.4)	
III	191 (61.8)	5 (55.6)	
IV	29 (9.4)	0	
Fracture type by AO/OTA classification			0.473
A1	147 (47.6)	3 (33.3)	
A2	155 (50.2)	5 (55.6)	
A3	7 (2.2)	1 (11.1)	
Basicervical component	150 (48.5)	8 (88.9)	**0.019**
Anterior cortex comminution	44 (14.2)	6 (66.7)	**0.001**
GT comminution	164 (53.1)	7 (77.8)	0.185
Time from admission to surgery (days)	2.6 ± 2.4	2.3 ± 0.9	0.732
Anesthesia (general: spinal)	263: 46	7: 2	0.545
Operation time (min)	64.1 ± 25.9	72.2 ± 31.4	0.361
Lag screw position in anteroposterior plane			0.888
Superior	6 (1.9)	0 (0)	
Center	156 (50.5)	5 (55.6)	
Inferior	147 (47.6)	4(44.4)	
Lag screw position in lateral plane			0.590
Anterior	66 (21.3)	2 (22.3)	
Center	181 (58.6)	4 (44.4)	
Posterior	62 (20.1)	3 (33.3)	
TAD (mm)	13.6 ± 3.6	14.3 ± 3.1	0.539
Cal-TAD (mm)	19.9 ± 4.2	20.5 ± 5.0	0.649
Quality of reduction			0.276
Good	237 (76.7)	5 (55.6)	
Acceptable	68 (22.0)	4 (44.4)	
Poor	4 (1.3)	0	
Reduction status			0.906
Anatomical	226 (73.1)	6 (66.7)	
Extramedullary	53 (17.2)	2 (22.2)	
Intramedullary	30 (9.7)	1 (11.1)	
Sliding distance (mm)	4.1 ± 3.6	18.3 ± 2.9	**0.001**

Data were presented by number (%) of patients or mean ± standard deviation.

ASA, American Society of Anesthesiologists; GT, greater trochanter; TAD, tip-to-apex distance; Cal, calcar.

In the comparative analysis between the non-basicervical group and the basicervical group, there were significant differences in age and BMD among the demographic data and in the presence of comminution of the anterior cortex and GT among the radiographic data. Patients in the basicervical group were older (p = 0.003) and had lower BMD (p = 0.010). In addition, more patients in the basicervical group showed comminution of the anterior cortex (21.7% vs. 9.9%, p = 0.004) and GT (63.7% vs. 44.1%, p = 0.001) on preoperative 3-D CT. Eventually, the basicervical group developed more fixation failures (8/157, 5.1%) including two cases of cut-outs, whereas there was only one case of excessive sliding in the non-basicervical group (1/161, 0.6%) (Table 4). On multiple logistic regression, low BMD (OR 1.32, p = 0.040) and comminution of the GT (OR 1.79, p = 0.027) were predictors of trochanteric fractures with a basicervical fracture component.

**Table 4:** Comparison of demographic, clinical, and radiologic data between non-basicervical and basicervical fracture groups.

Variables	Non-basicervical group (n = 161)	Basicervical group (n = 157)	*p*-value
Age (years)	78.4 ± 11.2	81.8 ± 8.8	**0.003**
Gender (female: male)	108: 53	113: 44	0.343
BMI (kg/m^2^)	22.2 ± 3.2	22.1 ± 3.9	0.889
BMD (T-score)	−2.9 ± 1.1	−3.2 ± 0.9	**0.010**
ASA score			0.159
II	54 (33.6)	39 (24.8)	
III	91 (56.5)	105 (66.9)	
IV	16 (9.9)	13 (8.3)	
Fracture type by AO/OTA classification			0.397
A1	72 (44.7)	78 (49.7)	
A2	83 (51.5)	77 (49.3)	
A3	6 (3.8)	0	
Anterior cortex comminution	16 (9.9)	34 (21.7)	**0.004**
GT comminution	71 (44.1)	100 (63.7)	**0.001**
Time from admission to surgery (days)	2.8 ± 2.8	2.4 ± 1.9	0.137
Anesthesia (general: spinal)	137:24	133:24	0.925
Duration of operation (min)	63.5 ± 27.9	65.3 ± 23.9	0.535
Lag screw position in AP plane			0.255
Superior	5 (3.1)	1 (0.6)	
Center	79 (49.1)	82 (52.2)	
Inferior	77 (47.8)	74 (47.2)	
Lag screw position in lateral plane			0.548
Anterior	36 (22.4)	32 (20.4)	
Center	89 (55.2)	96 (61.1)	
Posterior	36 (22.4)	29 (18.5)	
TAD (mm)	13.0 ± 3.3	14.2 ± 3.9	0.058
Cal.-TAD (mm)	19.5 ± 4.1	20.3 ± 4.2	0.063
Quality of reduction			0.636
Good	119 (73.9)	123 (78.3)	
Acceptable	40 (24.8)	32 (20.4)	
Poor	2 (1.3)	2 (1.3)	
Reduction status			0.659
Anatomical	121 (75.2)	111 (70.7)	
Extramedullary	26 (16.1)	29 (18.5)	
Intramedullary	14 (8.7)	17 (10.8)	
Time to union (weeks)	13.8 ± 8.8	13.2 ± 8.6	0.611
Sliding distance (mm)	4.5 ± 3.9	4.7 ± 4.3	0.685
Failure cases	1 (0.6)	8 (5.1)	**<0.001**
Cut-out	0	2	
Excessive collapse (>15 mm)	1	6	

Data were presented by number (%) of patients or mean ± standard deviation.

ASA, American Society of Anesthesiologists; AP, anteroposterior; TAD, tip-to-apex distance; Cal, calcar.

## Discussion

Because the CM nail has shown a clear advantage over the compression hip screw, the indications of CM nailing have greatly broadened^3–7^. These expanded indications have led to increased use of the CM nail for almost all trochanteric hip fractures, including in cases with basicervical fracture patterns^26–28^. However, fixation failures such as cut-out still occur owing to poor bone quality and an unstable fracture pattern despite the technical advances in implant designs. Among several comparable CM nails for the treatment of trochanteric hip fractures, the Gamma nail and PFNA are commonly used, which have reported cut-out rates ranging from 1.85% to 6.7%^29,30^. More recently, the Gamma3 nail with U-Blade lag screw was introduced to improve the stability against rotation and cut-out^31^. However, there is little information on the surgical outcomes of this nail in a large cohort of patients with trochanteric hip fractures. Therefore, we aimed to investigate the surgical outcomes of AO/OTA 31 A1-3 trochanteric fractures treated with Gamma3 nail with U-Blade lag screw and to evaluate the risk factors associated with fixation failures such as cut-out.

To increase the holding power for the proximal fragment and to decrease rotational instability leading to fixation failures such as cut-out in unstable trochanteric fractures, various types of CM nails have been designed, such as a helical blade for the firm fixation of the proximal fragment. An additional U-Blade lag screw for the Gamma3 nail was introduced to provide rotational stability to the proximal fragment. This U-Blade has a spreading effect that increases the surface in cranio-caudal direction, leading to higher resistance to failure and subsequently improving cut-out resistance, even in osteoporotic bone^18,31^. In addition, Gamma3 nail has an additional device called a fragment control clip, which allows the insertion of temporary anti-rotation pin into the femoral head to prevent rotation of the proximal fragment during lag screw placement. In unstable trochanteric fractures, especially along with a rotationally unstable short basicervical fracture component, the proximal fragment is likely to rotate with subsequent reduction loss during lag screw placement. Therefore, this additional device may be very available in these fractures.

As a result of these advantages, the current study showed that the overall cut-out rate of Gamma3 nail with U-Blade lag screw was 0.6% (2/309), which is much lower than the reported cut-out rates of comparable CM nails in the current literature. In addition, we observed a low complication rate (2.8%) including seven cases of excessive collapse that finally led to loss of reduction or non-union, whereas complication rates of around 7% were reported for the latest generation of the Gamma nails, including non-unions, nail breakage, distal screw breakage, secondary femoral fracture, and loss of reduction^30^. Although the appropriate TAD, calTAD, lag screw position, and the quality of reduction in most of our series and the surgeons’ expertise may have contributed to the favorable outcomes and low cut-out rate in this study, we believe that the use of an additional U-Blade lag screw, which was designed to improve rotational stability, played a role in improving the surgical outcomes with a low cut-out rate. Contrary to our study, Lang *et al*.^18^ reported that there was no statistically significant difference in the cut-out rate between the use of an additional U-Blade screw and the use of the standard screw of Gamma3 nail although the additional U-Blade lag screw reduced the cut-out rate compared with the standard lag screw (2.2% vs. 3.7%). In addition, they found that the cut-out rate in PFNA remained the smallest (1.2%) compared with Gamma3 with or without an additional U-Blade lag screw, although the PFNA showed significant migration within the femoral head^32^. However, they did not describe and compare the BMD, details of fracture patterns such the presence of a basicervical component and comminution of the anterior cortex, and quality of reduction, which can affect fixation failures such as cut-out. Further, they did not describe cut-through (the specific failure feature of PFNA), which is more likely to be caused by greater migration of the PFNA blade within the femoral head, as shown in a previous biomechanical study^33^. Finally, their cohort was relatively smaller than our cohort. Accordingly, we believe that their conclusions could not yet verify the greater effectiveness of an additional U-Blade lag screw of the Gamma3 nail than other comparable CM nails.

Several factors have been associated with failure of CM nailing for trochanteric hip fractures. Increased TAD or calTAD, inappropriate position of the lag screw within the femoral head, poor reduction or bone quality, unstable fractures along with anterior cortex or GT comminution including lateral wall fracture, and the presence of a basicervical component in complex fracture patterns were found to be the principal causes of implant cut-out and fixation failure^13,14,34^. Among these, modifiable factors such as TAD, calTAD, position of lag screw, and reduction quality were controlled, to some extent, and relatively appropriate in most of our series; thus they showed no significant differences between the union group and the failure group. In these conditions, non-modifiable factors such as fracture pattern had an important effect on fixation failures including cut-out. Watson *et al*.^15^ also reported that all fixation failures after CM nailing for basicervical trochanteric fractures occurred in patients with appropriate TAD (<25 mm) and anatomic or nearly anatomic reduction and suggested that factors other than the surgical technique were probably responsible for the failures. Bojan *et al*.^13^ reported that a basicervical fracture pattern is one of the three variables associated with a high risk of screw cut-out. Ciufo *et al*.^14^ found that a basicervical fracture component as well as lateral wall fracture along with GT comminution are risk factors associated with cut-out after CM nailing in pertrochanteric fractures. Meanwhile, Carr^9^ suggested that a relatively intact anterior and medial cortex should be accurately reduced for a more stable construct in unstable pertrochanteric fractures. Accordingly, if there is comminution in the anterior cortex, cortical contact and reduction can be obtained only in the medial cortex in these fractures. Therefore, the risk of rotational instability of the proximal fragment and fixation failure may increase, especially when accompanied by a basicervical fracture component or a posteromedial fragment. Our findings also showed that fracture patterns such as a basicervical fracture component and anterior cortex comminution were more frequently present in the failure group and were the main factors affecting the fixation failure, in agreement with previous studies. Therefore, the ideal implant for these fractures should have greater holding power for the proximal fragment and maintain its rotational stability during bone healing. On the basis of the results of the present study, we believe that the Gamma3 nail with U-Blade lag screw can provide a solution for these issues, to some extent.

In the literature as well as in our study, the presence of a basicervical fracture component was a risk factor associated with fixation failures such as cut-out in trochanteric hip fractures^13–15^. However, there is little information on the predictable factors associated with the occurrence of a basicervical fracture component in trochanteric fractures. The present study showed that osteoporosis and GT comminution including the lateral wall are predictable factors associated with a basicervical fracture component in trochanteric fractures. Poor bone quality due to osteoporosis in elderly patients with trochanteric fractures is more likely to cause fracture comminution including the GT and lateral wall and subsequently a basicervical fracture component, which subsequently increases the risk of fixation failure along with poor bone quality. Accordingly, these fracture patterns should be confirmed on preoperative 3-D CT. Further, it should be kept in mind that these patterns can more frequently develop in elderly patients with more severe osteoporosis.

The present study has several limitations. First, this was a retrospective study performed in serially observed patients despite using prospectively compiled data. Second, there was no comparative group treated with other CM nails. However, we enrolled consecutive elderly patients aged ≥70 years who underwent CM nailing with Gamma3 nail with U-Blade lag screw and compared the union group and the failure group to determine the risk factors associated with fixation failure, among non-modifiable factors. Another limitation is the relatively short follow-up period. However, a long-term follow-up study is barely possible and has little clinical relevance in elderly patients, who have a short life expectancy and limited life activities.

Despite these limitations, our study demonstrated that Gamma3 nail with U-Blade lag screw is a satisfactory alternative that can improve the rotational stability of the proximal fragment and subsequently obtain favorable surgical outcomes with a low fixation failure rate. A major strength of this study is that it is, to our knowledge, the largest cohort study to report the surgical outcomes of Gamma3 nail with U-Blade lag screw in elderly patients with trochanteric hip fractures and the first study to analyze the predictable factors of basicervical trochanteric fractures confirmed on 3-D CT.

In conclusion, Gamma3 nail with U-Blade lag screw showed favorable results for trochanteric hip fractures, with a low cut-out rate (0.6%). However, fixation failure is more likely to occur in trochanteric fractures with a basicervical fracture component and anterior cortex comminution. Therefore, more caution is required in treating these fracture patterns even with this nail.

## Data Availability

The data that support the findings of this study are available from the corresponding author upon reasonable request.
